# Abnormal uterine artery remodelling in the stroke prone spontaneously hypertensive rat

**DOI:** 10.1016/j.placenta.2015.10.022

**Published:** 2016-01

**Authors:** Heather Y. Small, Hannah Morgan, Elisabeth Beattie, Sinead Griffin, Marie Indahl, Christian Delles, Delyth Graham

**Affiliations:** Institute of Cardiovascular and Medical Sciences, College of Medical, Veterinary and Life Sciences, University of Glasgow, Glasgow, UK

**Keywords:** SHRSP, Uterine artery, Vascular remodelling, Hypertension

## Abstract

**Introduction:**

The stroke prone spontaneously hypertensive rat (SHRSP) is an established model of human cardiovascular risk. We sought to characterise the uteroplacental vascular response to pregnancy in this model and determine whether this is affected by the pre-existing maternal hypertension.

**Methods:**

Doppler ultrasound and myography were utilised to assess uterine artery functional and structural changes pre-pregnancy and at gestational day 18 in SHRSP (untreated and nifedipine treated) and in the normotensive Wistar-Kyoto (WKY) rat. Maternal adaptations to pregnancy were also assessed along with histology and expression of genes involved in oxidative stress in the placenta.

**Results:**

SHRSP uterine arteries had a pulsatile blood flow and were significantly smaller (70906 ± 3903 μm^2^ vs. 95656 ± 8524 μm^2^ cross-sectional area; p < 0.01), had a significant increase in contractile response (57.3 ± 10.5 kPa vs 27.7 ± 1.9 kPa; p < 0.01) and exhibited impaired endothelium-dependent vasorelaxation (58.0 ± 5.9% vs 13.9 ± 4.6%; p < 0.01) compared to WKY. Despite significant blood pressure lowering, nifedipine did not improve uterine artery remodelling, function or blood flow in SHRSP. Maternal plasma sFLT-1/PlGF ratio (5.3 ± 0.3 vs 4.6 ± 0.1; p < 0.01) and the urinary albumin/creatinine ratio (1.9 ± 0.2 vs 0.6 ± 0.1; p < 0.01) was increased in SHRSP vs WKY. The SHRSP placenta had a significant reduction in glycogen cell content and an increase in *Hif1α*, *Sod1* and *Vegf*.

**Discussion:**

We conclude that the SHRSP exhibits a number of promising characteristics as a model of spontaneous deficient uteroplacental remodelling that adversely affect pregnancy outcome, independent of pre-existing hypertension.

## Highlights

• Deficient uterine artery remodelling and uteroplacental blood flow in SHRSP pregnancy.
• Impact on maternal sFlt-1/PlGF ratio, litter size and placental gene expression.
• Nifedipine treatment does not improve remodelling and blood flow parameters.

## 1 Introduction

In human pregnancy, the cardiovascular (CV) system undergoes major adaptations in response to the developing fetus. A central change in the CV system is a steady increase of 40–50% in blood volume [1]. The resulting increase in cardiac output is accommodated by angiogenesis [2] and, predominantly, the adaptation of existing vasculature in order to stabilise blood pressure by reducing total peripheral resistance [3]. In particular, the remodelling of the uteroplacental vasculature plays a critical role in sustaining an adequate blood flow at the maternal–placental interface, thus providing the developing fetus with appropriate nutrients and oxygen. Deficiencies of the maternal uterine vasculature have been shown to be present in women with gestational hypertension [4], pre-eclampsia [5], fetal growth restriction [6] and recurrent pregnancy loss [7]. These conditions significantly contribute to maternal and fetal morbidity and mortality worldwide. Despite the impact of these pregnancy-related complications, there is a lack of understanding in the underlying pathophysiological mechanism of how and why the uteroplacental vasculature does not remodel sufficiently. The ability to study these underlying mechanisms of abnormal remodelling in human uteroplacental vasculature during pregnancy is limited and the use of small animal models has contributed greatly to this field. In particular, the rat shows a similar maternal vascular response to pregnancy [8] and exhibits the same haemochorial placental type as humans [9]. However, currently, there are no models which exhibit spontaneous deficient uterine artery remodelling accompanied by reduced uteroplacental blood flow without pharmacological, transgenic or surgical intervention.

The incidence of cardiovascular disease (CVD) in women of child-bearing age is increasing [10]. Despite a similar percentage of pregnancies being affected by pre-existing CVD (3–5%) [10], [11], [12] as some forms of pregnancy-specific hypertension such as pre-eclampsia; it has received relatively less research attention. Deficiencies in pregnancy-related vascular remodelling may be related to pre-existing CV risk as the adaptation of the CV system can be likened to a stress test causing these risk factors to be manifested clinically as pregnancy induced hypertension [13]. The stroke prone spontaneously hypertensive rat (SHRSP) is an established model of genetic predisposition to CVD and mimics many of the human traits observed in CVD. These rats are characterised by spontaneous hypertension and a higher rate of cardiovascular complications compared to the control strain; the Wistar Kyoto (WKY) rat [14]. Previous studies have briefly characterised pregnancy in the SHRSP [15] and have indicated that placental amino acid transport is deficient in these animals [16] however the response of the uterine vasculature to pregnancy in the SHRSP has not been studied. We hypothesized that pre-existing maternal CV risk in SHRSP would result in deficient remodelling of the uteroplacental vasculature in response to pregnancy. We aimed to characterise how SHRSP and WKY responded to pregnancy by analysing the structure and function of the uterine arteries before and during pregnancy as well as characterising the effects this may have on maternal and placental adaptation and fetal outcome. Furthermore, we carried out an intervention study in SHRSP only, using nifedipine to distinguish whether deficiencies in uterine vascular remodelling present in this model were dependent upon the presence of pre-existing hypertension.

## 2 Materials and methods

### 2.1 Animals

Female SHRSP and WKY were recruited to the study at 7 weeks of age. All animals were housed under controlled lighting (from 0700 to 1900 h) and temperature (21 ± 3 °C) and received a normal diet (rat and mouse No.1 maintenance diet, Special Diet Services). All animal procedures were approved by the Home Office according to the Animals (Scientific Procedures) Act 1886 (Project License 60/4286). All females were age-matched and time mated at 12 weeks of age (±4 days). Virgin animals were used at 15 weeks of age. Day 0 of pregnancy was defined as the day that a coital plug was observed indicative of successful mating having taken place.

For vascular studies, animals were split into two sub-groups where one was used to measure blood pressure by radiotelemetry and allowed to progress to parturition (untreated WKY, untreated SHRSP and nifedipine treated SHRSP; n = 6 in each group) and the other was used for ultrasound and sacrificed at gestational day (GD) 18 for *ex vivo* pressure and wire myography (untreated WKY, untreated SHRSP and nifedipine treated SHRSP; n = 6 in each group). For biochemical measurements and placental histology, untreated pregnant SHRSP and WKY rats (n = 8 in each group) were sacrificed at GD 18. For additional fetal and placental measurements untreated SHRSP (n = 4) and WKY rats (n = 4) were harvested at GD 14 and 20.

### 2.2 Nifedipine treatment of SHRSP

7 week old SHRSP began nifedipine treatment at 25 mg/kg/day administered in two doses: a 10 mg/kg/day dose mixed in a 1 ml aliquot of baby food and a 15 mg/kg/day dose in drinking water in order to maintain lowered blood pressure throughout the 24 h period. Stock solutions of nifedipine in drinking water were prepared in ethanol and diluted to the appropriate concentration with no more than a 0.8% final ethanol concentration.

### 2.3 Blood pressure measurement

Blood pressure from 7 to 10 weeks was measured using tail cuff plethysmography [17]. A radiotelemetry transmitter (calibration accurate within ± 6 mmHg) was implanted at 10 weeks into untreated WKY, untreated SHRSP and nifedipine treated SHRSP (n = 6) as previously described [18]. Systolic and diastolic blood pressure, heart rate and activity were then monitored using the Dataquest system (Data Sciences International).

### 2.4 Ultrasound and Doppler analysis of the uterine arteries

Uterine artery Doppler waveform recordings were used to assess *in vivo* uteroplacental blood flow in untreated SHRSP, nifedipine treated SHRSP and untreated WKY rats. Rats were lightly anaesthetized throughout the procedure at approximately 1.5% isoflurane in oxygen. Rats were imaged trans-abdominally using an Acuson Sequoia c256 ultrasound imager fitted with a 15-MHz linear array transducer. Peak systolic velocity (PSV) and end diastolic velocity (EDV) was measured from 6 consecutive cardiac cycles. Resistance index (RI) (RI = [PSV−EDV]/PSV) and S/D ratio (PSV/EDV) were calculated.

### 2.5 Wire and pressure myography of the uterine arteries

Virgin and pregnant animals were age matched at the point of sacrifice (GD 18). Uterine arteries were harvested from the same area of the horn (i.e. closer to the vagina than the ovary), and which contained the most fetuses. Arteries were not harvested from a horn that had less than four fetuses. Arteries were dissected in calcium free physiological salt solution (PSS) solution. Sections of uterine artery (4.8–5.2 μm in length) were mounted on the wire myograph (AD Instruments), normalized and subject to a wake up procedure as previously described [19]. To establish the vessel's contractile response, noradrenaline was added at the following increasing concentrations: 1 × 10^−9^, 3 × 10^−9^, 1 × 10^−8^, 3 × 10^−8^, 1 × 10^−7^, 3 × 10^−7^, 1 × 10^−6^, 3 × 10^−6^, 1 × 10^−5^ and 3 × 10^−5^ M. To determine the vessel's endothelial dependent relaxation response, vessels were pre-constricted with 3 × 10^−5^ M noradrenaline followed by the addition of carbachol at the following increasing concentrations: 1 × 10^−8^, 3 × 10^−8^, 1 × 10^−7^, 3 × 10^−7^, 1 × 10^−6^, 3 × 10^−6^ and 1 × 10^−5^ M. The pressure myograph system (Danish Myo Technology) was set up and equilibrated according to manufacturer's instructions. Vessels were maintained at 37 °C and 95% O_2_ and 5% CO_2_ throughout the experiment. Vessels were subject to a pressure curve at: 10, 20, 40, 60, 80, 100 and 110 mmHg. Measurements of internal (D_i_) and external diameter (D_e_) were taken after 5 min at each pressure. Wall thickness was calculated as [(D_e_ − D_i_)/2]. Cross sectional area was calculated as $\left[\left(\pi /4\right)\times \left({\text{D}}_{\text{e}}^{2}-{\text{D}}_{\text{i}}^{2}\right)\right]$. Wall strain was calculated as [D_i_/D_e_]. Wall stress was calculated as [(133.4 × pressure mmHg × D_i_)/2 × wall thickness] where 1 mmHg = 133.4 dyn/cm^2^. Wall stress was then divided by 10^6^ to give x10^6^ dyn per cm^2^.

### 2.6 Maternal, placental and fetal characterisation

Water intake and urine output pre-pregnancy and at different gestational time points were monitored by housing the animals in a metabolic cage for 24 h. Animals were acclimatised to the metabolic cage prior to use. Urine and extracted plasma were stored at −80 °C until use. Animals were sacrificed at GD 18 when blood was collected by cardiac puncture and fetal and placental tissue weighed. Placental tissue was harvested and either dissected into the decidua, junctional zone, labyrinth zone and chorionic plate, snap frozen in liquid nitrogen and stored at −80 °C until use or fixed in 10% formalin for 24 h and processed for histology. Accurate dissection of the placenta was confirmed using qPCR markers for the various zones (Supp. Fig. 1).

### 2.7 Gene expression

RNA extraction from tissues was performed using the miRNeasy mini kit (Qiagen) according to manufacturer's instructions. Total RNA concentration was determined by Nanodrop (Thermo Fisher Scientific) and stored at −80 °C until use. Gene expression RT-PCR was performed using the Taqman^®^ Reverse Transcription Kit (Applied Biosystems) according to manufacturer's instructions with 1 μg RNA input. The reaction was run on a Multi Block System Satellite 0.2 Thermo Cooler (Thermo Fisher Scientific) on the following settings: 25 °C 10 min, 48 °C 30 min, 95 °C 5 min. Quantitative PCR (qPCR) of *Hif1α*, *Sod1* and *Vegf* was performed using Taqman^®^ Universal Mastermix (Applied Biosystems) and relevant Taqman^®^ probe (Applied Biosystems). Gene expression protocol was run on an ABI PRISM 7900HT PCR system at the following settings: 95 °C, 15 min; followed by 40 cycles of 95 °C, 15 s; 60 °C, 1 min. Ct values were analysed using the 2(-delta delta Ct) method, with dCt indicating normalisation to the housekeeper, *Gapdh*. Primer details are provided in Supp. Table 1.

### 2.8 Histology

Tissues were prepared for histology using standard paraffin process methods. 5 μm sections were mounted. Mid-sagittal sections of placenta were stained using standard haematoxylin and eosin to assess structure. Perl's Prussian blue stain was used to assess free blood [20]. Briefly, 4% ferrocyanide solution and 4% acidified water were mixed immediately before incubating slides for 45 min then counter-stained with 1% neutral red. Sections were also stained using periodic-acid Schiff (PAS) stain to assess glycogen cell content. Briefly, equal 10 min incubations in 1% periodic acid followed by Schiff stain (Sigma) were used. Staining and layer measurements were quantified using ImageJ software [21] by two operators blinded to animal identification. Size of the placental layers was expressed as a percentage of the total pixels and positive staining was expressed as the percentage of positive pixels across the tissue section.

### 2.9 Biochemical measurement

Plasma soluble fms-like tyrosine kinase-1 (sFLT-1) and placental growth factor (PlGF) were measured using commercially available kits (R&D Systems) according to manufacturer's instructions. Albumin and creatinine were measured using commercially available kits (Roche) analysed using a Roche Cobas C311 Analyser.

### 2.10 Statistical analysis

All data are represented as mean ± standard error of the mean (SEM). Relevant statistical information is given in the figure legend.

## 3 Results

### 3.1 The SHRSP show deficient uterine artery remodelling in response to pregnancy

**Fig. 1:**
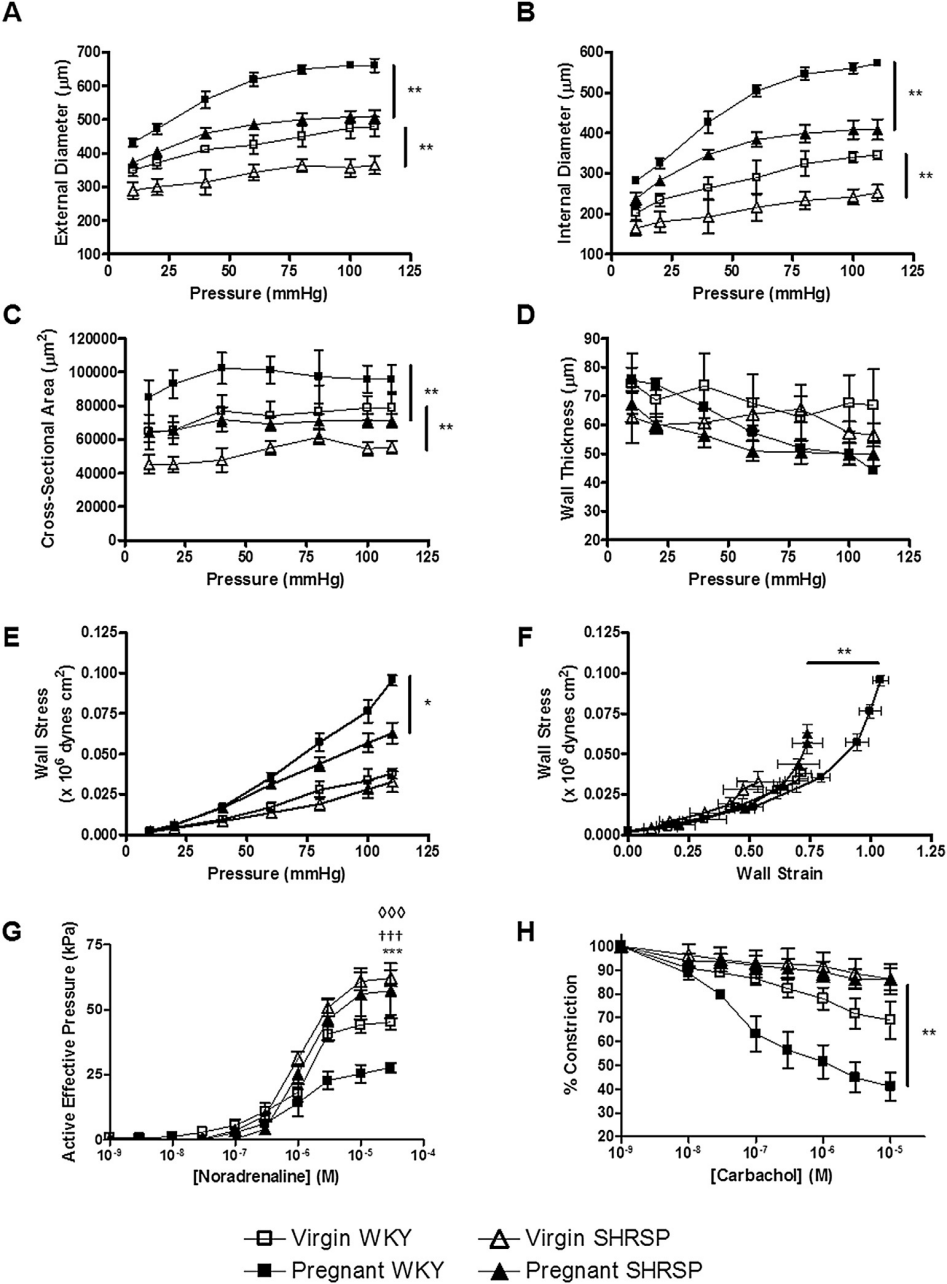
The SHRSP exhibits deficient pregnancy-associated uterine artery remodelling compared to the WKY. Isolated uterine artery structure was assessed using pressure myography to measure external (A) and internal (B) diameter, cross-sectional area (C) and wall thickness (D) in female virgin WKY, pregnant WKY (gestational day 18), virgin SHRSP and pregnant SHRSP (gestational day 18) (n = 4–6). External and internal diameter as well as cross-sectional area was significantly decreased in the pregnant SHRSP compared to the WKY (**p < 0.01). Wall stress (E) was significantly decreased in pregnant SHRSP compared to the pregnant WKY. The stress/strain curve (F) was shifted to the right upon pregnancy in both strains but was significantly decreased in the pregnant SHRSP. Isolated uterine artery function was also measured using wire myography in the same animals. Maximum response to noradrenaline (G) was significantly decreased in virgin vs. pregnant WKY (†††p < 0.001). Virgin SHRSP had a significantly increased maximum response to noradrenaline relative to virgin WKY (◊◊◊ p < 0.001). Pregnant SHRSP vessels did not have a significantly decreased response to noradrenaline vs. virgin SHRSP. Pregnant SHRSP vessels had a significantly increased maximum response to pregnant WKY vessels (***p < 0.001). The EC50 values for noradrenaline were not significantly different between groups (data not shown). Furthermore, endothelium-dependent vasorelaxation to carbachol (H) was significantly impaired in both virgin and pregnant SHRSP (**p < 0.01 vs. WKY). Data analysed by comparing area under the curve values using one way ANOVA and Tukey's *post-hoc* test.

Uterine artery structure from virgin and pregnant (GD 18) SHRSP and WKY rats were examined using pressure myography. WKY uterine arteries demonstrated a pregnancy dependent (virgin vs GD 18) increase in diameter and cross sectional area (Fig. 1A–C). SHRSP virgin uterine arteries were significantly smaller in diameter and cross-sectional area than virgin WKY arteries (δ area under the curve: internal diameter 872.44 vs. 1064.25; external diameter 791.67 vs. 961.79). SHRSP uterine arteries also exhibited a significant pregnancy dependent change (with an increase in diameter and cross-sectional area (virgin vs GD 18)); however this change was not to the same extent as in WKY arteries. SHRSP uterine arteries at GD 18 demonstrated a significant decrease in both external (Fig. 1A) and internal diameter (Fig. 1B) as well as cross sectional area (Fig. 1C). There was no significant change in wall thickness (Fig. 1D) between strains or between virgin and pregnant animals in either strain. Wall stress/pressure relationship (Fig 1E) was similar between strains in virgin animals, however at GD 18 there was a significant decrease in wall stress in the SHRSP relative to pregnant WKY. Furthermore, the stress/strain relationship (Fig. 1F) in the uterine arteries was shifted to the right in WKY pregnant animals indicating an increase in vessel distensibility. This rightward shift was less pronounced in SHRSP uterine arteries indicating increased stiffness relative to the WKY.

*Ex vivo* wire myography in uterine arteries from pregnant WKY demonstrated a significant reduction in maximum contractile response to noradrenaline (Fig. 1G) and a significant increase in endothelium-dependent relaxation to carbachol (Fig. 1H) when compared to arteries from virgin WKY rats. The uterine arteries of virgin SHRSP showed an increased maximum contractile response to noradrenaline, and a reduced endothelium-dependent relaxation to carbachol, relative to virgin WKY (Fig. 1G–H). The contractile response in uterine arteries from SHRSP at GD 18 was not significantly different from pre-pregnancy (Fig. 1G) and showed no significant increase in carbachol-mediated relaxation (Fig. 1H).

### 3.2 Nifedipine treatment significantly lowers blood pressure in the SHRSP

**Fig. 2:**
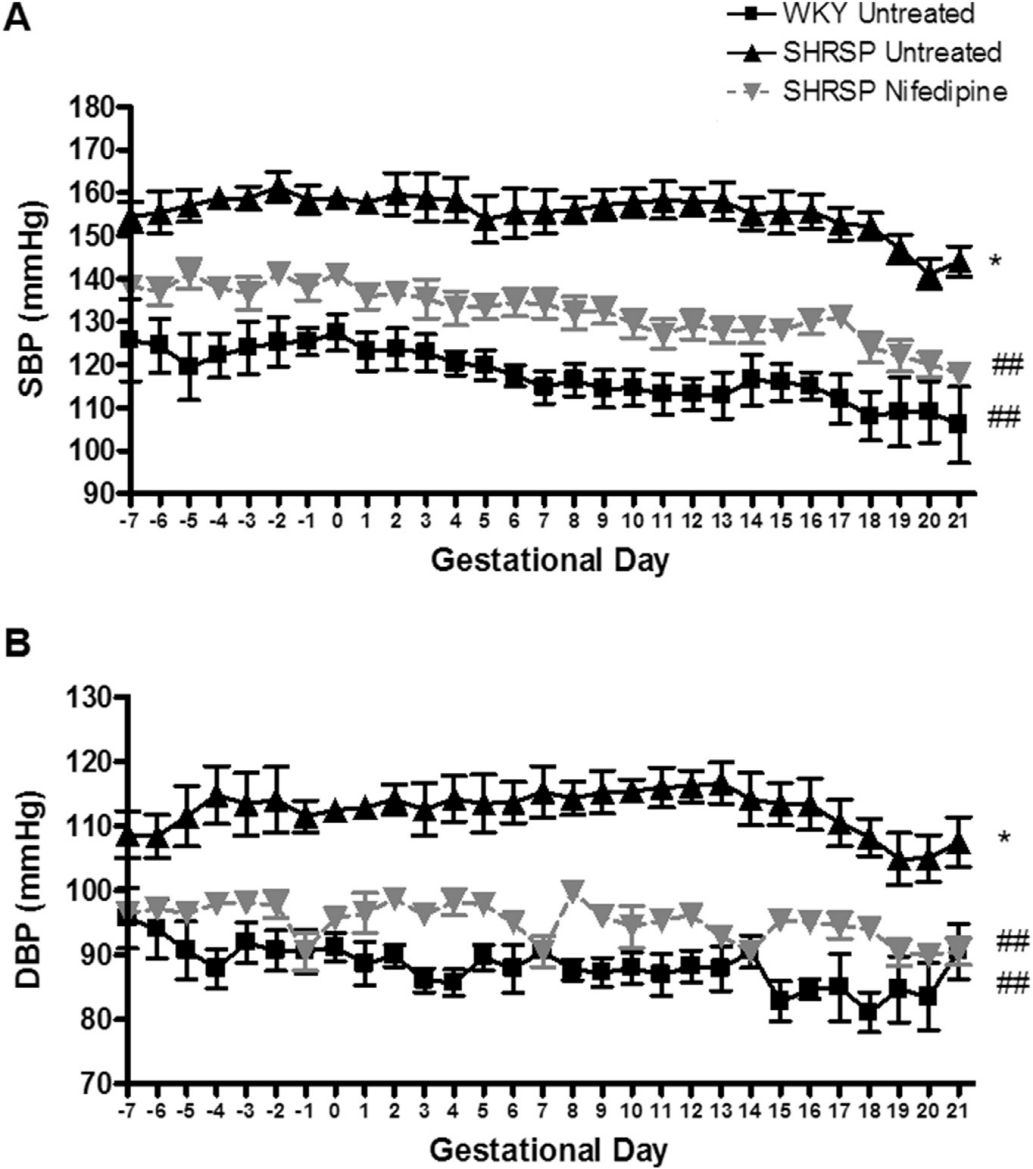
Nifedipine significantly inhibits blood pressure elevation in the SHRSP. Systolic (SBP) (A) and diastolic (DBP) (B) blood pressure was monitored in untreated WKY, untreated SHRSP and nifedipine treated SHRSP (n = 6) using radiotelemetry before day 0 and during pregnancy (gestational day 0 – day 21). SHRSP had significantly increased blood pressure compared to WKY (*p < 0.05). Nifedipine treatment significantly reduced SHRSP blood pressure (##p < 0.01 vs. SHRSP). Data analysed by comparing area under the curve values using one way ANOVA and Tukey's *post-hoc* test.

SHRSP were hypertensive before and during pregnancy (average pregnancy BP 155/112 mmHg), therefore we sought to examine whether the observed deficient uterine artery remodelling was dependent upon the presence of elevated blood pressure. Nifedipine (25 mg/kg/day), was administered in SHRSP from 7 weeks of age to prevent the development of hypertension prior to and during pregnancy. Blood pressure measurement by tail cuff plethysmography (Supp. Fig. 2) followed by radiotelemetry probe implantation at 10 weeks of age confirmed that nifedipine treated SHRSP were not hypertensive at any point during the study. Moreover, blood pressure levels in nifedipine treated SHRSP (average pregnancy BP 132/95 mmHg) were not significantly different from the normotensive WKY rats (average pregnancy BP 117/88 mmHg) (Fig. 2).

There was a general trend for systolic blood pressure to gradually decrease in early pregnancy followed by a small increase at the beginning of the final week of gestation before decreasing again prior to delivery in both the WKY and nifedipine treated SHRSP groups. This pattern was not observed in the SHRSP where blood pressure remained elevated and relatively stable over the course of pregnancy until GD 16 when it also decreased until delivery. In particular, the change in systolic blood pressure over the course of pregnancy (Supp. Fig. 3) was at its greatest between the SHRSP and WKY from GD 10 to 14. Heart rate was not significantly different between groups but activity was significantly decreased in both SHRSP and nifedipine treated SHRSP relative to the WKY over the course of pregnancy (Supp. Fig. 4)

### 3.3 Nifedipine treatment does not improve uterine artery structure or function in the SHRSP

**Fig. 3:**
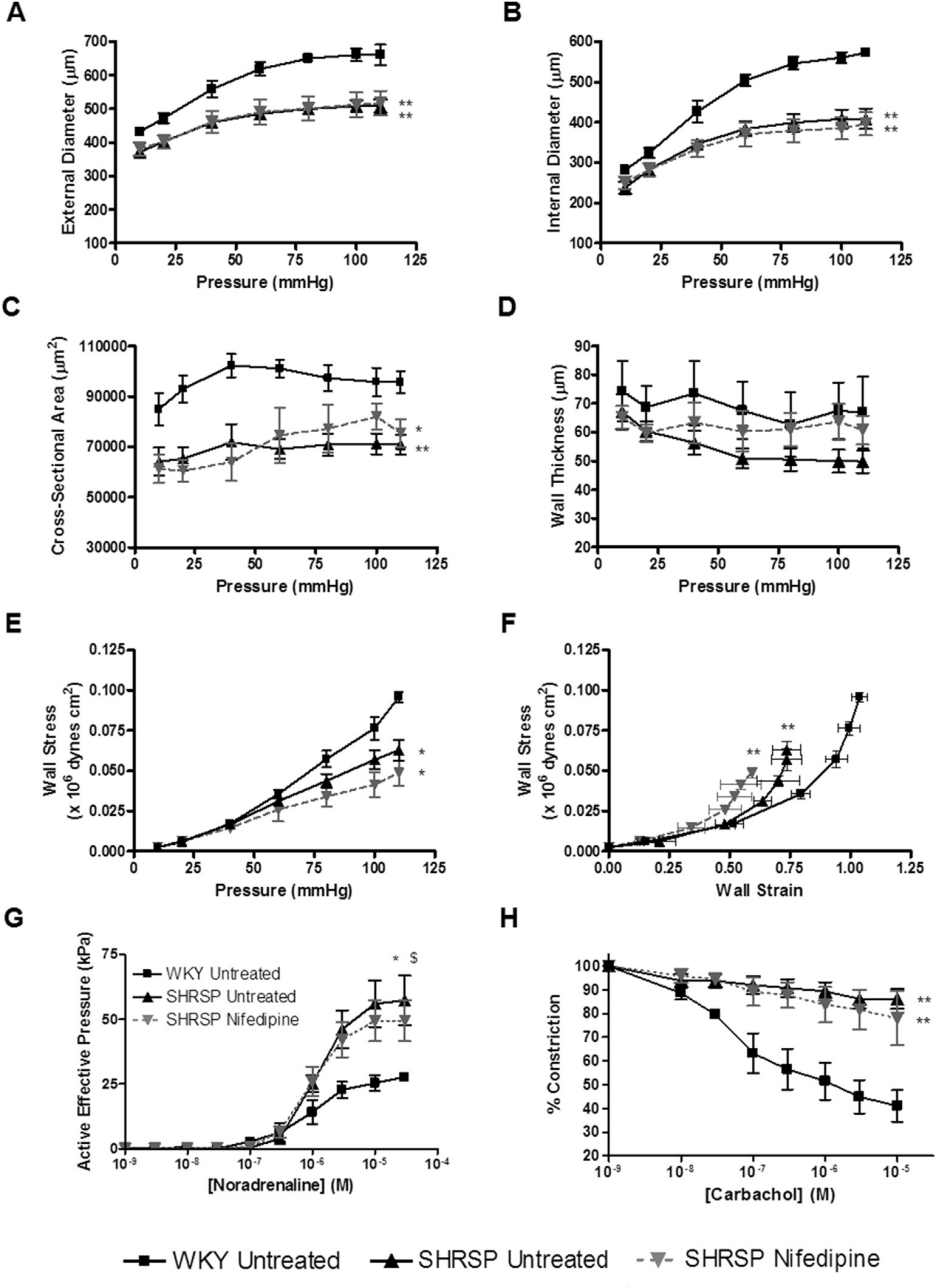
Nifedipine treatment does not improve uterine artery structure or function in the SHRSP. Isolated uterine artery structure was assessed using pressure myography to measure external (A) and internal (B) diameter, cross-sectional area (C) and wall thickness (D) in pregnant (gestational day 18) untreated WKY, untreated SHRSP and nifedipine treated SHRSP (n = 6). External and internal diameter as well as cross-sectional area was significantly decreased in both untreated and nifedipine treated SHRSP (*p < 0.05 vs. WKY; **p < 0.01 vs. WKY). Wall stress (E) was significantly reduced and stress/strain relationship shifted to the left (F) in both untreated and nifedipine treated SHRSP. Isolated uterine artery function was also measured using wire myography in the same animals. Maximum response to noradrenaline (G) was significantly increased in both virgin and pregnant SHRSP (*p < 0.05 SHRSP vs. WKY, $ p < 0.05 nifedipine vs. WKY). The EC50 values for noradrenaline were not significantly different between groups (data not shown). Furthermore, endothelium-dependent vasorelaxation to carbachol (H) was significantly impaired in both untreated and nifedipine treated SHRSP (**p < 0.01 vs. WKY). Data analysed by comparing area under the curve values using one way ANOVA and Tukey's *post-hoc* test.

Uterine artery structure from pregnant (GD 18) untreated WKY, untreated SHRSP and nifedipine treated SHRSP were examined using pressure myography. WKY uterine arteries were significantly larger in diameter and cross-sectional area than arteries from SHRSP and nifedipine treated SHRSP at GD 18. Vessels from nifedipine treated SHRSP showed similar external (Fig. 3A) and internal (Fig. 3B) diameter and cross-sectional area (Fig. 3C) compared to untreated SHRSP, therefore, uterine artery diameter in the SHRSP was not improved when hypertension was ameliorated using nifedipine. The passive mechanical properties of the uterine arteries (Fig. 3E–F) were also not significantly improved by nifedipine treatment. Nifedipine treatment did not significantly alter the maximum contractile response to noradrenaline (Fig. 3G) or the endothelium-dependent relaxation response to carbachol (Fig. 3H) in SHRSP uterine arteries.

### 3.4 Nifedipine treatment does not improve uterine artery blood flow or litter size in the SHRSP

**Fig. 4:**
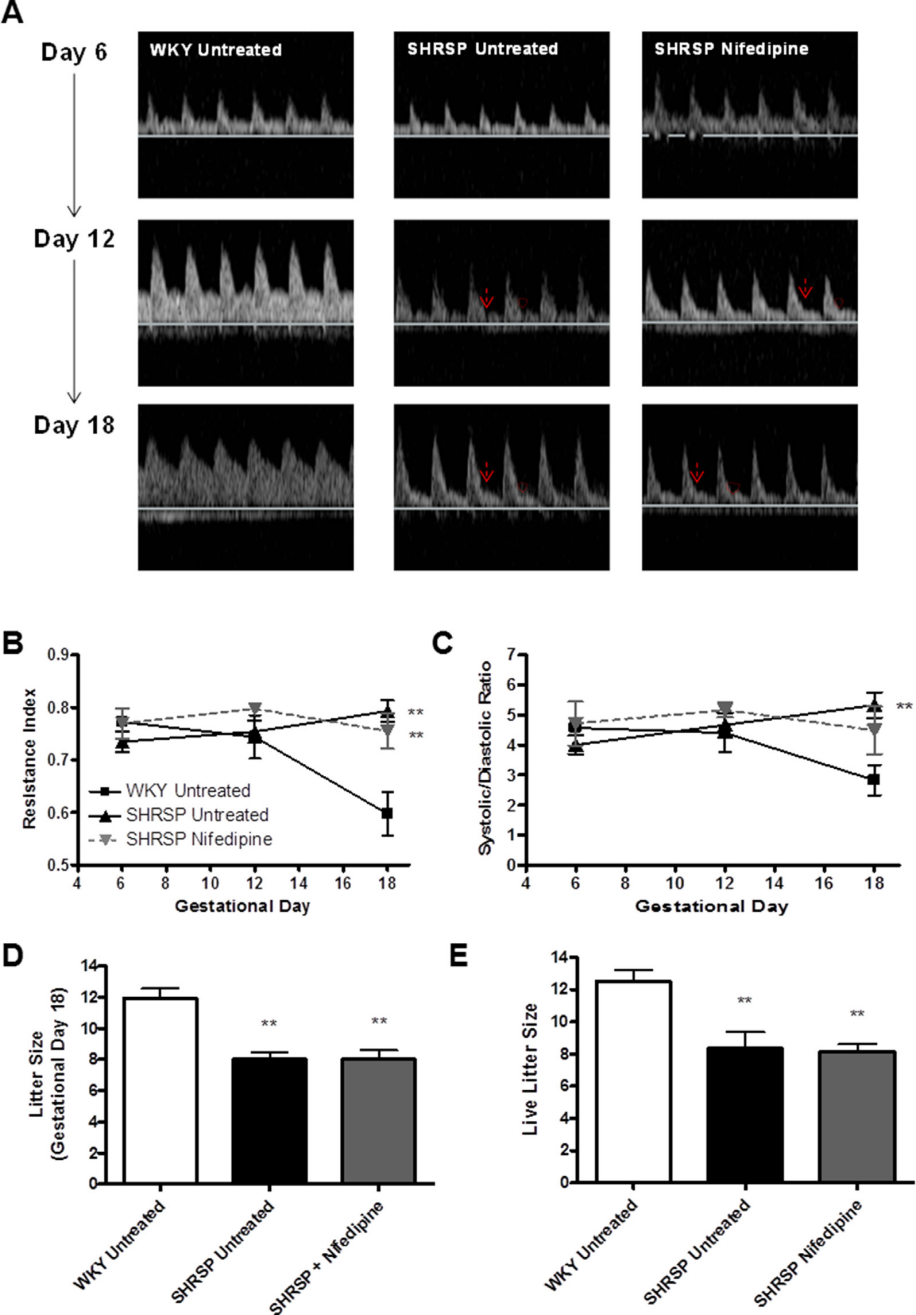
Nifedipine treatment does not improve *in vivo* uterine artery blood flow or reduced litter size in the SHRSP. Uterine artery blood flow was assessed using echo Doppler analysis at gestational day 6, 12 and 18 in pregnant untreated WKY, untreated SHRSP and nifedipine treated SHRSP (n = 6). Representative Doppler traces are shown in A. Resistance index (B) and systolic/diastolic ratio (C) were significantly decreased in WKY at gestational day 18 compared to both untreated and nifedipine treated SHRSP (**p < 0.01 vs. WKY). Litter size, counted at gestational day 18 (D) and live-born neonates at parturition (E), were significantly reduced in both untreated and nifedipine treated SHRSP (**p < 0.01 vs. WKY). Statistical significance determined using one-way ANOVA with Tukey's *post-hoc* test.

The *ex vivo* myography data (Fig. 1) showed that there was significantly reduced uterine artery remodelling in the SHRSP in response to pregnancy. We sought to examine the functional consequences on *in vivo* uteroplacental blood flow using Doppler ultrasound (Fig. 4A). WKY animals showed an increase in uterine artery blood flow with significantly reduced resistance index (Fig. 4B) and systolic/diastolic ratio (Fig. 4C) by GD 18. In contrast, the SHRSP did not show a significant change in resistance index (Fig. 4B) or systolic/diastolic ratio (Fig. 4C) over the course of pregnancy, resulting in the maintenance of a pulsatile blood flow profile (Fig. 4A). Notching, an indication of increased resistance of blood flow toward the placenta, was also present in the SHRSP (Fig. 4A). Nifedipine treated SHRSP did not show an improvement in the Doppler profile concurrent with the uterine artery myography data. Litter size at both GD 18 and parturition, was significantly decreased in the SHRSP compared to WKY (Fig. 4D–E). Furthermore, the incidence of resorptions was also increased in untreated SHRSP (66%) compared to WKY (36%*)* and nifedipine treated SHRSP (25%) (Table 1). The litter size was not improved by nifedipine treatment.

### 3.5 SHRSP have an altered maternal response to pregnancy

**Fig. 5:**
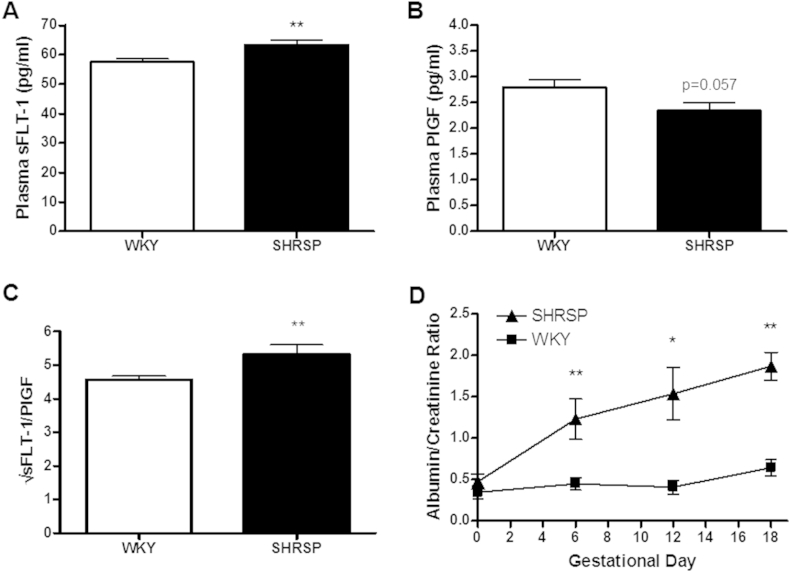
Markers of hypertensive pregnancy are increased in the SHRSP. 24 h urine samples were collected at gestational days 6, 12 and 18 and plasma was collected at gestational day 18 from pregnant untreated SHRSP and WKY (n = 8). Plasma sFLT-1 (A) was increased in the SHRSP and PlGF (B) showing a trend for decrease in the SHRSP. The plasma sFLT-1 to PlGF ratio was significantly increased in the SHRSP (C) (**p < 0.01 vs. WKY). Urine albumin to creatinine ratio (D) was significantly increased in the SHRSP over the course of pregnancy (*p < 0.05 vs. WKY; **p < 0.01 vs. WKY). Statistical significance determined using Student's t-test.

In SHRSP rats at GD 18, maternal plasma sFLT-1 was significantly increased (Fig. 5A) whereas PlGF exhibited a trend towards a decrease (Fig. 5B). Concurrently, sFLT-1/PlGF ratio was modestly but significantly increased in SHRSP (Fig. 5C). Maternal urine was collected over the course of pregnancy. Albumin/Creatinine ratio (ACR) was similar between strains pre-pregnancy (Fig. 5D). However, as pregnancy progressed the ACR significantly increased in the SHRSP. In contrast, ACR values in WKY rats remained relatively stable over the course of pregnancy. Pregnancy dependent and independent maternal weight gain was significantly reduced in SHRSP at GD 18 relative to WKY (Supp. Table 2 and Supp. Fig. 5).

### 3.6 SHRSP exhibit placental abnormalities

We found no evidence of fetal or placental weight changes between the two strains at GD 18 and both demonstrated similar fetal and placental growth trajectories from GD 14 to GD 20 (Supp. Fig. 6). The head:body ratio was also similar in WKY and SHRSP fetuses at GD 20 (Supp. Fig. 6).

**Fig. 6:**
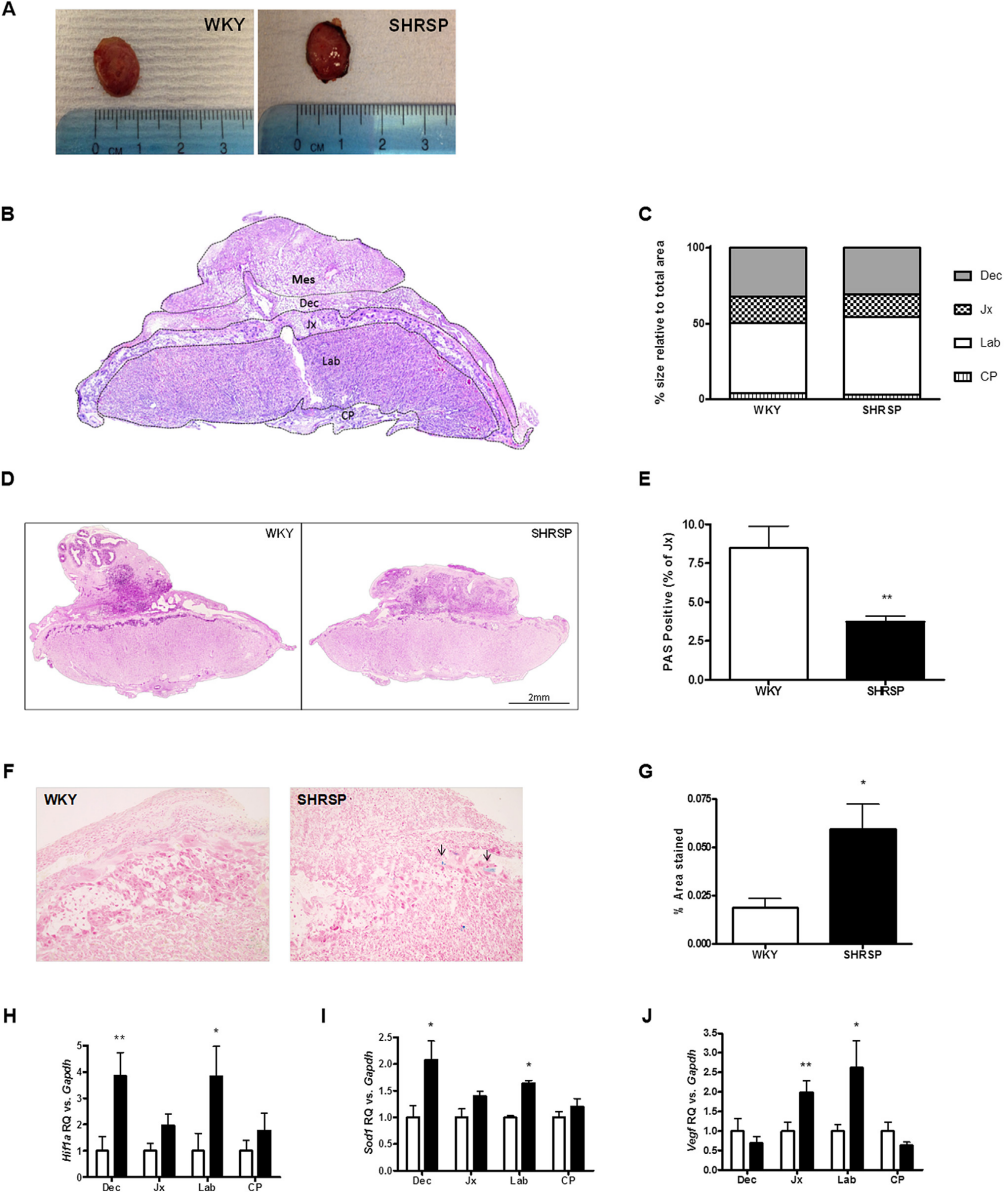
Placental dysfunction in the SHRSP. Once dissected from the fetus the placentas were photographed maternal side facing up and dark areas confined to the edges of the placenta were seen in SHRSP only (A). A representative H&E mid-saggital section is shown and the mesometrial triangle (Mes), decidua (Dec), junctional zone (Jx), labyrinth zone (Lab) and chorionic plate (CP) are labelled (B). There was no significant difference in size between the layers of the placenta (C). Periodic acid-Schiff stain was used to quantify glycogen cell content (n = 6) which was significantly decreased in the SHRSP (D–E). Perl's Prussian blue stain was used to identify free blood (n = 6) and was significantly increased in the SHRSP (F–G). Placentae from SHRSP and WKY (n = 8) were dissected into the main layers of the placental unit: decidua (Dec), junctional zone (Jx), labyrinth zone (Lab) and chorionic plate (CP). Genes of interest were assessed in these zones separately; the SHRSP showed an increase in *Hif1a* (H) and *Sod1* (I) expression in both the decidua and labyrinth whilst *Vegf* (J) mRNA was upregulated in the junctional zone and labyrinth. Statistical significance determined using Student's t-test (**p < 0.01 vs. WKY; *p < 0.05 vs. WKY).

Despite no change in placental weight, visual inspection of the tissue on dissection (Fig. 6A) identified a darkened area of potential haemorrhage/necrosis within the outer region of the junctional zone in the majority of SHRSP placenta which was not evident in the WKY. Haematoxylin and eosin staining showed that there were no significant size differences between the placental layers of the two strains (Fig. 6B–C). However, periodic acid Schiff (PAS) stain revealed that the SHRSP have a significantly reduced proportion of PAS positive glycogen cells in the junctional zone of the placenta compared to WKY (Fig. 6D–E)., There was no significant difference in staining in the other zones of the placenta (Data not shown). Perl's Prussian blue staining revealed a significant increase in free blood principally localised in the junctional zone of the placenta in the SHRSP compared to the WKY (Fig. 6F–G). Furthermore, gene expression of *Hif1a* (Fig. 6H), *Sod1* (Fig. 6I) and *Vegf* (Fig. 6J) were significantly up-regulated in the labyrinth zone of the SHRSP placenta at GD 18. SHRSP also showed significantly up-regulated *Hif1a* and *Sod1* expression in the decidua, and *Vegf* was significantly increased in the junctional zone.

## 4 Discussion

This study aimed to characterise uterine artery remodelling during pregnancy in the setting of maternal CVD using the SHRSP rat model. Furthermore, an intervention study was completed using nifedipine in the SHRSP rat to determine whether the presence of pre-existing hypertension played a role in the observed deficient uterine artery remodelling.

SHRSP exhibit deficient uterine artery remodelling in response to pregnancy compared to the WKY control strain, which to our knowledge, is the first evidence that pre-existing CV risk can affect the remodelling of the uteroplacental vasculature. One strength of our study is the characterisation of pregnant uterine arteries relative to non-pregnant uterine arteries, a control which has been identified as lacking in other studies [22]. Our results show that non-pregnant SHRSP have smaller uterine arteries with endothelial dysfunction compared to the WKY. Both SHRSP and WKY uterine arteries increase in diameter and cross sectional area in response to pregnancy but demonstrate no change in wall thickness. This is indicative of outward hypertrophic remodelling [23], and is also observed in human uterine arteries in response to pregnancy [24]. Measurement of the passive mechanical wall properties indicates that pregnancy causes an increase in uterine arterial distensibility in normal rats, however SHRSP vessels demonstrate an increased stiffness compared to WKY. This is similar to the uterine artery properties of other rat models of uteroplacental insufficiency [25] and in women with hypertensive complications during pregnancy [26]. In contrast to the pregnancy dependent structural changes that were observed in both rat strains, there was a reduction in the contractile ability of the uterine artery and an increased endothelium-dependent relaxation response observed only in the WKY, suggesting that SHRSP uterine arteries have an abnormal functional response to pregnancy.

The blood pressure profile in early pregnancy in WKY and SHRSP rats shows similarities to humans. In healthy human pregnancy and in WKY rats, blood pressure decreases towards the middle stage of pregnancy [27]. In contrast, women who develop pregnancy induced hypertension (PIH) do not experience this early decrease in blood pressure [27], similar to that observed in the SHRSP rat. In particular, the SHRSP rat exhibits the greatest blood pressure elevation relative to the WKY from GD 10 to 14. This coincides with the critical period of development and maturation of the rodent placenta around day 12. The main difference in blood pressure profile between human and both rodent strains is that blood pressure decreases towards delivery in rats whereas in humans blood pressure returns to pre-pregnancy levels. This reduction in blood pressure observed from GD 18 has been recorded in other pregnancy studies using rat models [28]. It has been hypothesised that a “hypotensive factor” is increased in the circulation of SHRSP to induce this fall in blood pressure towards parturition [29].

The altered uterine artery remodelling in the SHRSP rat coincides with a deficient uteroplacental blood flow. These observations are not improved by treatment with the clinically relevant drug, nifedipine; even though systemic blood pressure is corrected, indicating that deficient uterine artery remodelling in the SHRSP is not dependent upon the presence of chronic hypertension. Similarly, nifedipine treatment at 22–24 weeks gestation in women who went on to develop pre-eclampsia has been shown to significantly reduce blood pressure but did not improve abnormal uterine artery blood flow as assessed by Doppler ultrasound [30]. Specifically, Doppler analysis of the SHRSP uterine arteries showed a reduced diastolic volume and the presence of notching. These are clinical indicators of abnormal uterine artery remodelling in humans [31], [32]. Our work supports previous findings in the related spontaneously hypertensive rat (SHR) in which placental perfusion was reduced by 26% compared to normotensive controls [33], furthermore this was not improved by nifedipine treatment [34]. Uteroplacental blood flow has also recently been shown to be reduced during pregnancy in salt sensitive Dahl rats, a salt-inducible hypertensive rat strain [35].

We found that the deficient uterine artery remodelling and reduced uteroplacental perfusion in the SHRSP rat had no significant effect on fetal and placental weight but were accompanied by abnormalities in maternal and placental physiology. During pregnancy, the human biomarker for PIH, sFLT-1/PlGF ratio [32] was significantly increased in maternal plasma in the SHRSP rat relative to the WKY. We have found that SHRSP dams do not show proteinuria when measured by bicinchoninic acid assay (BCA) (data not shown); however ACR steadily increases over the course of pregnancy in the SHRSP despite a similar ACR to the WKY rat pre-pregnancy. This indicates that there may be pregnancy-related maternal kidney dysfunction in these animals. With respect to the fetus, previous work has shown growth restricted fetuses from the SHR strain; however in this previous study the animals were sacrificed at a later gestational age (GD 20) [36]. We did not observe any fetal growth restriction, at GD 18 or GD 20 in the SHRSP, indicating that fetal growth was not affected by the hypertensive conditions in this model. We did, however, observe a reduction in litter size consistent with previous findings in the SHRSP [15] and an increase in the number of resorptions observed per litter in the SHRSP which has not been previously reported. Our hypothesis is that the reduced litter size and increased number of resorptions observed in SHRSP rats are due to sub-optimal uteroplacental blood flow resulting from deficient uterine artery remodelling. However, at this stage, we have not determined whether these changes are a cause or effect of the altered remodelling.

We have shown evidence of glycogen cell loss in the SHRSP placenta, specifically in the junctional zone. As no other alterations were observed in the other placental layers (i.e. decidua) we conclude that this is not a result of the migration of the glycogen cells but a depletion in the SHRSP placenta compared to WKY. The reduction in uteroplacental perfusion in the SHRSP may cause the placenta to utilise glycogen stores prematurely relative to the WKY. The SHRSP had visible placental abnormalities in the darkened outer region of the junctional zone. Perl's Prussian blue staining confirmed there was a larger proportion of free blood in the SHRSP junctional zone; however this was not typical of placental haemorrhage that has been observed in other studies [37]. We hypothesise that these darkened areas could be necrosis due to insufficient perfusion; however this is yet to be confirmed. Characterisation of the placenta using a candidate approach for those genes known to be involved in human hypertensive pregnancy [38], [39], [40] revealed that the SHRSP placenta had expression patterns consistent with a hypoxic environment. Particularly, both *Hif1a* and *Sod1* were up-regulated in the maternal vascular compartment of the decidua. However, whether the expression of these genes is a cause or effect of abnormal uteroplacental perfusion is yet to be determined.

The strengths of the SHRSP as a model of hypertensive pregnancy are that it is a spontaneous model which requires no genetic, pharmacological or surgical intervention making it easier to use with increased reproducibility. Alongside this, the deficient uterine artery remodelling and high blood pressure in SHRSP are present during the critical time of placental development, in contrast to many surgically induced models such as the RUPP [41], where the placenta is already fully developed when the surgical intervention to reduce uteroplacental blood flow is performed (i.e. between GD 14–15). We propose that the pregnant SHRSP rat is a relevant addition to existing animal models of abnormal uteroplacental blood flow and will be complimentary to models such as the RUPP. The SHRSP rat also has pre-existing maternal CV risk and, as the incidence of CVD is increasing among women of child bearing age [42], this model will become increasingly clinically relevant. Recently, a large human study has shown that maternal hypertension during early pregnancy as opposed to late pregnancy, has a greater impact upon blood pressure in the offspring [43]. This highlights the strength of the SHRSP as a model since hypertension is present in the early stages of pregnancy. Additionally, a recent meta-analysis examining pregnancy in women with chronic hypertension has highlighted the prominence of this problem in Western society and the increased risk of adverse outcomes for both mother and child [44]. The SHRSP model will allow important pre-clinical investigations of the underlying mechanisms of abnormal vascular adaption to pregnancy and allow us to examine the respective contribution of pre-existing maternal CV risk factors and chronic hypertension to this process.

In summary, we have shown that pregnant SHRSP rats have deficient uterine artery remodelling and that this is independent of chronic hypertension. Deficient uterine artery remodelling is a common underlying cause of a number of human pregnancy disorders. In our model, we have provided evidence that the altered remodelling has functional consequences for uteroplacental blood flow and impacts upon the mother as well as the placenta. The underlying causative factors of the spontaneous deficient uterine artery remodelling in the SHRSP rat are still to be determined but we believe that they will be important in highlighting the underling mechanisms in human deficient uteroplacental perfusion.

## Conflicts of interest

None to declare.

## Figures and Tables

**Table 1:** Litter number and percentage resorptions in WKY, SHRSP and nifedipine treated SHRSP.

	WKY untreated	Untreated SHRSP	SHRSP nifedipine
Litter number (GD 18)	12 ± 0.66	9 ± 0.72*	8 ± 0.85*
Litter number (parturition)	12 ± 0.71	8 ± 1.02**	8 ± 0.46**
Resorption frequency	36%	66%	25%

Resorption frequency was defined as expressing the number of fetuses resorbed as a percentage of total litter size (live + resorptions) within a single pregnancy in a single animal. *p < 0.05 vs WKY untreated, **p < 0.01 vs WKY untreated.

## References

[1] Clapp J.F., Seaward B.L., Sleamaker R.H., Hiser J. (1988). Maternal physiologic adaptations to early human pregnancy. Am. J. Obstet. Gynecol..

[2] Zygmunt M., Herr F., Munstedt K., Lang U., Liang O.D. (2003). Angiogenesis and vasculogenesis in pregnancy. Eur. J. Obstet. Gynecol. Reprod. Biol..

[3] Osol G., Moore L.G. (2014). Maternal uterine vascular remodeling during pregnancy. Microcirculation.

[4] Pijnenborg R., Anthony J., Davey D.A., Rees A., Tiltman A., Vercruysse L. (1991). Placental bed spiral arteries in the hypertensive disorders of pregnancy. Br. J. Obstet. Gynaecol..

[5] Brosens I.A., Robertson W.B., Dixon H.G. (1972). The role of the spiral arteries in the pathogenesis of preeclampsia. Obstet. Gynecol. Annu..

[6] Todros T., Sciarrone A., Piccoli E., Guiot C., Kaufmann P., Kingdom J. (1999). Umbilical Doppler waveforms and placental villous angiogenesis in pregnancies complicated by fetal growth restriction. Obstet. Gynecol..

[7] Quenby S., Nik H., Innes B., Lash G., Turner M., Drury J. (2009). Uterine natural killer cells and angiogenesis in recurrent reproductive failure. Hum. Reprod..

[8] Osol G., Cipolla M. (1993). Pregnancy-induced changes in the three-dimensional mechanical properties of pressurized rat uteroplacental (radial) arteries. Am. J. Obstet. Gynecol..

[9] Soares M.J., Chakraborty D., Karim Rumi M.A., Konno T., Renaud S.J. (2012). Rat placentation: an experimental model for investigating the hemochorial maternal-fetal interface. Placenta.

[10] Seely E.W., Ecker J. (2014). Chronic hypertension in pregnancy. Circulation.

[11] Bateman B.T., Bansil P., Hernandez-Diaz S., Mhyre J.M., Callaghan W.M., Kuklina E.V. (2012). Prevalence, trends, and outcomes of chronic hypertension: a nationwide sample of delivery admissions. Am. J. Obstet. Gynecol..

[12] Sibai B.M. (2002). Chronic hypertension in pregnancy. Obstet. Gynecol..

[13] Sattar N., Greer I.A. (2002). Pregnancy complications and maternal cardiovascular risk: opportunities for intervention and screening?. BMJ.

[14] Okamoto K., Yamamoto K., Morita N., Ohta Y., Chikugo T., Higashizawa T. (1986). Establishment and use of the M-strain of stroke-prone spontaneously hypertensive rat. J. Hypertens..

[15] Yamada N., Kido K., Tamai T., Mukai M., Hayashi S. (1981). Hypertensive effects on pregnancy in spontaneously hypertensive rats (SHR) and stroke-prone SHR (SHRSP). Int. J. Biol. Res. Pregnancy.

[16] Fuchi I., Higashino H., Noda K., Suzuki A., Matsubara Y. (1995). Placental Na+, K+ activated ATP-ase activity in SHRSP in connection with pregnancy induced hypertension and intra-uterine growth retardation. Clin. Exp. Pharmacol. Physiol. Suppl..

[17] Bunag R.D., Butterfield J. (1982). Tail-cuff blood-pressure measurement without external preheating in awake rats. Hypertension.

[18] Anderson N.H., Devlin A.M., Graham D., Morton J.J., Hamilton C.A., Reid J.L. (1999). Telemetry for cardiovascular monitoring in a pharmacological study: new approaches to data analysis. Hypertension.

[19] Spiers A., Padmanabhan N. (2005). A guide to wire myography. Methods Mol. Med..

[20] Bancroft J.D., Stevens A. (1996). Theory and Practice of Histological Techniques.

[21] Schneider C.A., Rasband W.S., Eliceiri K.W. (2012). NIH Image to ImageJ: 25 years of image analysis. Nat. Methods.

[22] McCarthy F.P., Kingdom J.C., Kenny L.C., Walsh S.K. (2011). Animal models of preeclampsia; uses and limitations. Placenta.

[23] Osol G., Mandala M. (2009). Maternal uterine vascular remodeling during pregnancy. Physiol. Bethesda.

[24] Palmer S.K., Zamudio S., Coffin C., Parker S., Stamm E., Moore L.G. (1992). Quantitative estimation of human uterine artery blood flow and pelvic blood flow redistribution in pregnancy. Obstet. Gynecol..

[25] Mazzuca M.Q., Tare M., Parkington H.C., Dragomir N.M., Parry L.J., Wlodek M.E. (2012). Uteroplacental insufficiency programmes vascular dysfunction in non-pregnant rats: compensatory adaptations in pregnancy. J. Physiol..

[26] Savvidou M.D., Kaihura C., Anderson J.M., Nicolaides K.H. (2011). Maternal arterial stiffness in women who subsequently develop pre-eclampsia. PLoS One.

[27] Hermida R.C., Ayala D.E., Mojon A., Fernandez J.R., Alonso I., Silva I. (2000). Blood pressure patterns in normal pregnancy, gestational hypertension, and preeclampsia. Hypertension.

[28] Scott J.N., Goecke J.C. (1984). Profile of pregnancy in young spontaneously hypertensive rats. Jpn. Heart J..

[29] Kubota T., Yamada T. (1981). Circulating hypotensive factor in pregnant spontaneously hypertensive rats. Clin. Exp. Pharmacol. Physiol..

[30] Cobellis L., De Luca A., Pecori E., Mastrogiacomo A., Di Pietto L., Iannella I. (2006). Mid-trimester fetal-placental velocimetry response to nifedipine may predict early the onset of pre-eclampsia. In Vivo.

[31] Papageorghiou A.T., Yu C.K., Nicolaides K.H. (2004). The role of uterine artery Doppler in predicting adverse pregnancy outcome. Best. Pract. Res. Clin. Obstet. Gynaecol..

[32] Verlohren S., Stepan H., Dechend R. (2012). Angiogenic growth factors in the diagnosis and prediction of pre-eclampsia. Clin. Sci. Lond..

[33] Ahokas R.A., Reynolds S.L., Anderson G.D., Lipshitz J. (1987). Uteroplacental blood flow in the hypertensive, term pregnant, spontaneously hypertensive rat. Am. J. Obstet. Gynecol..

[34] Ahokas R.A., Sibai B.M., Mabie W.C., Anderson G.D. (1988). Nifedipine does not adversely affect uteroplacental blood flow in the hypertensive term-pregnant rat. Am. J. Obstet. Gynecol..

[35] Gillis E.E., Williams J.M., Garrett M.R., Mooney J.N., Sasser J.M. (2015). The dahl salt sensitive rat is a spontaneous model of superimposed preeclampsia. Am. J. Physiol. Regul. Integr. Comp. Physiol..

[36] Johnston B.M. (1995). Fetal growth retardation and increased placental weight in the spontaneously hypertensive rat. Reprod. Fertil. Dev..

[37] Liu L., Zhao G., Fan H., Zhao X., Li P., Wang Z. (2014). Mesenchymal stem cells ameliorate Th1-induced pre-eclampsia-like symptoms in mice via the suppression of TNF-alpha expression. PLoS One.

[38] Rajakumar A., Brandon H.M., Daftary A., Ness R., Conrad K.P. (2004). Evidence for the functional activity of hypoxia-inducible transcription factors overexpressed in preeclamptic placentae. Placenta.

[39] Trollmann R., Amann K., Schoof E., Beinder E., Wenzel D., Rascher W. (2003). Hypoxia activates the human placental vascular endothelial growth factor system in vitro and in vivo: up-regulation of vascular endothelial growth factor in clinically relevant hypoxic ischemia in birth asphyxia. Am. J. Obstet. Gynecol..

[40] Vanderlelie J., Venardos K., Clifton V.L., Gude N.M., Clarke F.M., Perkins A.V. (2005). Increased biological oxidation and reduced anti-oxidant enzyme activity in pre-eclamptic placentae. Placenta.

[41] Li J., LaMarca B., Reckelhoff J.F. (2012). A model of preeclampsia in rats: the reduced uterine perfusion pressure (RUPP) model. Am. J. Physiol. Heart Circ. Physiol..

[42] Regitz-Zagrosek V., Gohlke-Barwolf C., Iung B., Pieper P.G. (2014). Management of cardiovascular diseases during pregnancy. Curr. Probl. Cardiol..

[43] Staley J.R., Bradley J., Silverwood R.J., Howe L.D., Tilling K., Lawlor D.A. (2015). Associations of blood pressure in pregnancy with offspring blood pressure trajectories during childhood and adolescence: findings from a prospective study. J. Am. Heart Assoc..

[44] Bramham K., Parnell B., Nelson-Piercy C., Seed P.T., Poston L., Chappell L.C. (2014). Chronic hypertension and pregnancy outcomes: systematic review and meta-analysis. BMJ.

