# Supplementary material for: Abnormal uterine artery remodelling in the stroke prone spontaneously hypertensive rat
Source: Placenta. 2016 Jan;37:34–44. doi: 10.1016/j.placenta.2015.10.022 (PMC4721537; doi:10.1016/j.placenta.2015.10.022)
Supplement: Supplementary file 1 [file mmc1.docx]

**ABNORMAL UTERINE ARTERY REMODELLING IN THE STROKE PRONE SPONTANEOUSLY HYPERTENSIVE RAT**

Heather Y SMALL _a_, Hannah MORGAN _a_, Elisabeth BEATTIE _a_, Sinead GRIFFIN _a_, Marie INDAHL _a_, Christian DELLES _a_, Delyth GRAHAM _a_

_a_ Institute of Cardiovascular and Medical Sciences, College of Medical, Veterinary and Life Sciences, University of Glasgow, Glasgow, UK.

**Short Title**: Abnormal Uterine Artery Remodelling in the SHRSP

**CORRESPONDENCE TO**: Heather Y. Small, Institute of Cardiovascular and Medical Sciences, College of Medical, Veterinary and Life Sciences, University of Glasgow, 126 University Place, Glasgow G12 8TA, UK.

E-mail: [h.small.1@research.gla.ac.uk](mailto:h.small.1@research.gla.ac.uk).


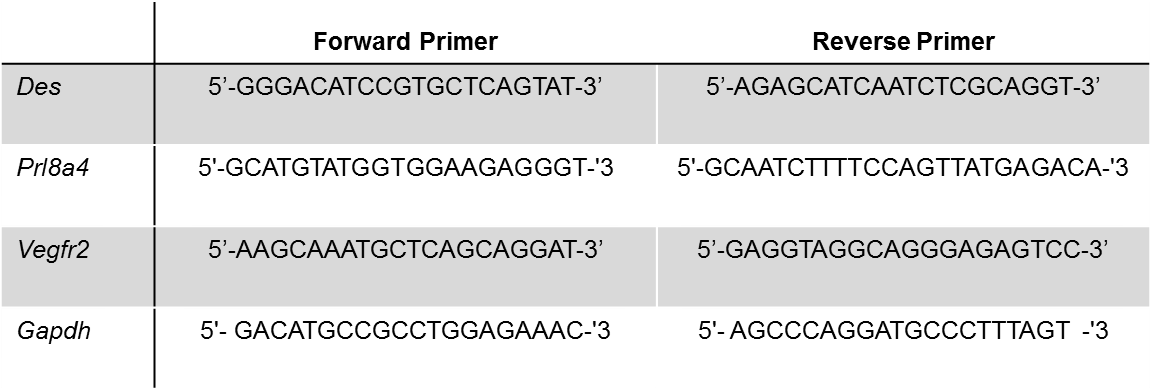


**Supplementary Table One: Primer sequences for primers used to assess accurate dissection of placental layers**


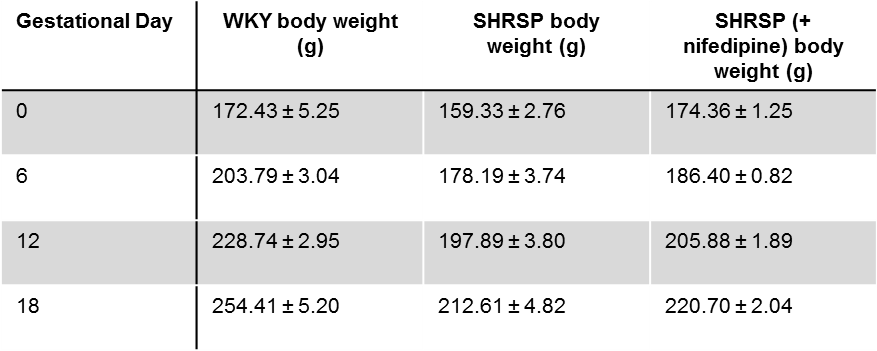


**Supplementary Table Two: Maternal body weight**

n=6 per group, ** p<0.01, *** p<0.005 vs. WKY

**A**

**C**

**B**

**Supplementary Figure One: mRNA expression of specific placental zone markers.** The placenta was dissected into four layers: Dec - Decidua; Jx – Junctional zone; Lab – Labyrinth zone; CP – Chorionic plate. Quantitative PCR (qPCR) was performed using PowerSybr^®^ reagent (Applied Biosystems and relevant Taqman^®^ probe (Applied Biosystems). Gene expression protocol was run on an ABI PRISM 7900HT PCR system at the following settings: 95 °C, 15 min; followed by 40 cycles of 95 °C, 15 s; 60 °C, 1 min followed by a 30 minute dissociation step to ensure primer specificity. Ct values were analysed using the 2(-delta delta Ct) method, with dCt indicating normalisation to the housekeeper, Gapdh. Primers are given in supplementary table 2. Desmin was most highly expressed in the decidual and junctional layers (A). Prl8a4 was most highly expressed in the junctional zone with almost no expression in other zones (B). Vegfr2 had increased expression in the predominantly vascular zones of the placenta (C); the decidua and the labyrinth. Significance determined using one-way ANOVA to compare different layers in the respective strains (n=3-5); **p<0.01 vs WKY; ***p<0.001 vs WKY; ##p<0.01 vs SHRSP; ###p<0.001 vs SHRSP.

**Supplementary Figure Two: Nifedipine treatment prevents SHRSP from developing hypertension from an early age.** Prior to radiotelemetry implantation at 10 weeks, systolic blood pressure was monitored using tail cuff plethysmography. This was to ensure that nifedipine treated SHRSP never became hypertensive at any point in the study. Untreated SHRSP (n=6) had a significantly increased systolic blood pressure at 9 weeks of age (** p<0.01 vs. nifedipine treated SHRSP). This increase was prevented by nifedipine treatment. Data analysed by comparing area under the curve values using Student’s t-test.

**A**

**B**

**Supplementary Figure Three: Blood pressure changes over gestation.** Telemetry data was adjusted by averaging the 7 days pre-pregnancy blood pressure measurements and subtracting this from gestational blood pressure changes. There is a similar diastolic blood pressure (DBP) trend throughout gestation in WKY, SHRSP and nifedipine treated SHRSP (n=6 in each group) (A). The systolic blood pressure (SBP) was increased in SHRSP between gestational day 10 and 14 (highlighted by red bracket), compared to WKY and the nifedipine treated SHRSP, however it followed the same overall pattern blood pressure decreasing throughout pregnancy (B).

**A**

**B**

**Supplementary Figure Four: Heart rate and activity changes prior to and during pregnancy.** WKY, SHRSP and nifedipine treated SHRSP had similar heart rates prior to and during gestation. SHRSP had significantly reduced activity compared to the WKY during pregnancy. This was not improved by nifedipine treatment. Day 0 indicates successful mating. * p<0.05 vs. WKY; n=6. Data was analysed using two-way ANOVA.

**A**

**B**

**Supplementary Figure Five: Pregnancy-dependent and independent maternal weight gain is reduced in the SHRSP and is not improved by nifedipine.** Maternal weight gain over the course of pregnancy (A) was significantly decreased in both the SHRSP and SHRSP + nifedipine groups. Data was analysed by comparing area under the curve values using one way ANOVA. Since this reduction in weight gain could be attributed to a reduction in litter size in these animals, the animals were weighed at GD 18 without the uteroplacental unit which we have referred to as “pregnancy independent” weight gain. Pregnancy independent weight gain was again significantly reduced in the SHRSP and SHRSP + nifedipine groups (B). Data was analysed using one way ANOVA.

**A**

**B**

**C**

**D**

**Supplementary Figure Six: Fetal and placental weight is not significantly different in the SHRSP and WKY.** Growth curves for fetal (A) and placental (B) weights show there is no significant change in fetal or placental weights at GD 14 (n=4 for both strains), 18 (n=8 for both strains) or 20 (n=4 for both strains). The fetal/placental (F:P) ratio (C) were not significantly different at GD 18 between the two strains and the head body ratio at GD 20 were also not significantly different (D).
